# A Geospatially Customizable Culturally Tailored Just-in-Time Adaptive Intervention for Violence-Affected People Living With HIV: Mixed Methods Acceptability, Feasibility, and User Experience Study

**DOI:** 10.2196/80705

**Published:** 2026-06-26

**Authors:** Simone J Skeen, Stephanie Tokarz, Rayna E Gasik, Ethan A Smith, Katherine P Theall, Gretchen A Clum

**Affiliations:** 1 Department of Social, Behavioral, and Population Sciences, Celia Scott Weatherhead School of Public Health and Tropical Medicine, Tulane University, 1440 Canal Street, New Orleans, LA, 70112, United States, 1 504-988-5388; 2 Connolly Alexander Institute for Data Science, Tulane University, New Orleans, LA, United States; 3 Department of Epidemiology, Celia Scott Weatherhead School of Public Health and Tropical Medicine, Tulane University, New Orleans, LA, United States

**Keywords:** mobile health, mHealth, HIV, traumatic stress, posttraumatic growth, coping, geospatial, just-in-time adaptive intervention, JITAI

## Abstract

**Background:**

Posttraumatic stress, along with comorbid mental health challenges and hazardous alcohol use, disproportionately affects people living with HIV. The drivers of these stressors are both intraindividual, rooted in early life adversity and firsthand violence exposures, and contextual, often place-based. Imparting effective coping skills and distinguishing between changeable and unchangeable stressors can improve stress management in the short term, with cascading effects on key HIV continuum of care end points, such as antiretroviral therapy adherence. However, problem- and emotion-based coping skills, delivered via traditional linear in-person group modalities, may falter in the moment. To address this, we adapted the evidence-based Living in the Face of Trauma intervention into an iOS- and Android-native app, featuring daily diary–triggered coping skills recommendations, self-guided Living in the Face of Trauma psychoeducational sessions, and a customizable geofencing function.

**Objective:**

This mixed methods study aimed to examine the acceptability, feasibility, and user experiences of NOLA (New Orleans, Louisiana) Gem, focusing on user interaction costs relative to geographic ecological momentary assessment (GEMA) alone and refining future optimization options.

**Methods:**

People living with HIV (N=32) were recruited across New Orleans and initially randomized 1:1 to treatment (NOLA Gem + GEMA) versus control (GEMA) for 21 days. Feasibility was assessed via enrollment and attrition rates. At the immediate postassessment, participants completed acceptability and usability measures and a brief structured usability interview. Analyses included descriptive statistics, bivariate logit modeling, and synergistic human–large language model deductive coding.

**Results:**

In total, 30 participants (n=22 in the GEMA + NOLA Gem treatment arm) completed the pilot, representing 94% (n=29) of baseline enrollees. Acceptability was very high across the board: 100% (n=30) of users considered NOLA Gem “very” or “somewhat” successful in addressing their daily lives, with 91% (n=28) endorsing increased calm and emotional well-being. In addition, 50% (n=11) of NOLA Gem users were “extremely likely” (Net Promoter Score=10/10) to recommend the app to friends. Eight (27%) GEMA and GEMA + NOLA Gem users reported privacy concerns. Eleven (50%) NOLA Gem users received geofencing alerts; perceptions of this feature’s helpfulness were mixed. No statistically significant sociodemographic or clinical predictors of disparate acceptability or increased privacy concerns were found. No additional frictions were evidenced by GEMA + NOLA Gem versus GEMA users. Qualitatively, NOLA Gem users praised the just-in-time mindfulness, breathing, problem-solving skills delivery, and broader stress control and self-insight benefits. A subset of users pointed out the burdensome length and sometimes inconvenient timing of the daily diaries. Recommendations for next-generation personalization included user-specific dynamic daily diary and geofencing prompt tailoring.

**Conclusions:**

Our small pilot study demonstrated high NOLA Gem acceptability and feasibility, as well as a rich and beneficial user experience among people living with HIV, with clear and actionable opportunities for improvement.

## Introduction

### Background

Posttraumatic stress is endured by an estimated 28% of people with HIV, globally [1]. Nearly two-thirds (64%) report symptoms consistent with diagnosable posttraumatic stress disorder (PTSD) [2]. The trauma burden can reach as high as 97.1%, with an average of 4.2 out of 10 recognized adverse childhood experiences (ACEs) on the ACEs Questionnaire, among women with HIV in clinical samples. Lifetime trauma has been linked, in a dose-response manner, to greater likelihoods of self-reported depressive and anxiety symptoms, as well as alcohol and illicit drug use [3]. Indeed, the origins of traumatic stress among many people with HIV in the United States are often attributable to multiple sources, including compounded early life adversities, economic hardship, and, in particular, firsthand violence exposures [4-6]. Brown et al [5], in South Carolina, reported 3 overarching components of traumatic life experiences among people with HIV: firsthand extreme violence and secondhand homicide and suicide exposures, physical and sexual assault, and accidents and natural disasters. Among people with HIV, these experiences can encompass childhood sexual abuse [4], racial trauma, incarceration [5], homelessness, rape, intimate partner and intrafamilial violence, and comorbid life-threatening illness [6].

Brezig et al [7], pointing to the disproportionate prevalence of violent (vs nonviolent) trauma among people with HIV, developed a syndemic theory of trauma alongside the acquisition, forward transmission, and later-life outcomes associated with HIV survivorship. Trauma, per the American Psychological Association, can manifest in heightened fear, vulnerability, rumination, disorientation, and dissociative tendencies [2]. Among some people with HIV, these symptoms are linked to anger, isolation, distrust [6,7], comorbid anxiety, depression, suicidal ideation [8,9], and hazardous drug and alcohol use [2,7].

Through a behavioral medicine lens, posttraumatic stress represents a crucial, robustly demonstrated, within-person determinant of antiretroviral therapy (ART) adherence [2-4]. Among women with HIV, acute trauma is linked to 4 times the odds of lapsed ART [7]. Within the Brown et al [5] sample, people with HIV who experienced past extreme violence–related or death-related trauma were 63% less likely (adjusted odds ratio 0.37, 95% CI 0.15-0.95) to demonstrate adherence than non–trauma-exposed participants; people with HIV exposed to any trauma were 58% less likely (adjusted odds ratio 0.42, 95% CI 0.21-0.86). Among Black gay and bisexual men in Baltimore, Maryland, and Jackson, Mississippi, 24% reported past-year physical, sexual, or psychological violence exposure, and recent and lifetime violence exposure was linked to ART nonadherence (adjusted prevalence risk ratio 1.59, 95% CI 1.25-2.01) [10]. Qualitatively similar effects, entangling lifetime trauma, mental health challenges, and substance use or misuse as drivers of poor ART adherence are consistently demonstrated across independent samples of people with HIV and US geographies [11-13], including New Orleans [14].

Narrowly individualistic perspectives on trauma among people with HIV may overlook important neighborhood-based sources of stress [15,16] and stressors that can change across time and space even within a single day [17,18]. In New Orleans, neighborhood-level alcohol outlet density has been linked to alcohol misuse among men with HIV and an increased likelihood of endorsing depressive symptoms among women with HIV [19]. GPS and daily diary data (from a very small feasibility sample; n=11) demonstrate that people with HIV spend an average of just 19% of their awake time within their own residential Census tracts, exposed to neighborhood crime rates ranging from 1 to 28 per 1000 population and alcohol outlet density rates of 0 to 22 per 1000 as they traversed, on average, 23 Census tracts [20]. Emerging evidence, typically captured via geographic ecological momentary assessment, shows that these dynamic place-based factors can affect mood and rumination among people with HIV in New Orleans [9,21].

Effective coping can interrupt traumatic stress and ART adherence lapses [22-25]. Specifically, *adaptive* coping, grounded in the coping framework of Lazarus and Folkman [26], distinguishes between “changeable” and “unchangeable” stressors. The former are approached via problem-focused strategies, such as structured problem-solving and effective communication skills, along with additional cognitive behavioral therapy–oriented strategies, while the latter are addressed via emotion-focused strategies, including cognitive restructuring, mindfulness meditation, and confronting irrational beliefs such as “all-or-nothing” thinking [23]. The Living in the Face of Trauma (LIFT) intervention by Sikkema et al [25], a Centers for Disease Control and Prevention “best evidence” intervention for improving coping with early life trauma stressors and HIV, as well as reducing trauma symptom burden, exemplifies this approach. Across a series of pilot studies [25] and fully powered randomized controlled trials [27], as well as an adaptation for women with HIV in Cape Town, South Africa [28], LIFT has demonstrated efficacy in reducing intrusive symptom burden, forward transmission risk behaviors, and avoidance and arousal PTSD symptoms, while improving ART adherence.

A 15-session, linear, in-person group intervention, LIFT, by definition, does not respond to momentary ecological stressors that people with HIV may encounter throughout the day. Just-in-time adaptive interventions (JITAIs), delivered via smartphone, and often reliant on ecological momentary assessment (EMA) inputs [29,30], can prompt users with brief psychoeducational and coping skills refreshers whenever necessary [24,31]. Similarly, geofencing interventions deliver intervention content triggered by spatial inputs, wherever necessary [32]. JITAIs have demonstrated preliminary efficacy in reducing ruminative episodes via cognitive behavioral therapy skills prompts and alcohol misuse among homeless adults in pilot studies [33,34], as well as robust efficacy profiles among adults transitioning from inpatient alcohol use disorder treatment [35], alongside a wide array of mental health endpoints [36,37]. Reported acceptability, feasibility, and engagement, including among sexually minoritized men living with HIV [38], are generally high [34,39-41], with many opportunities for incorporating prospective end user insights [42].

### Study Objectives

To our knowledge, no prior studies have examined the acceptability, feasibility, and broader user experience of a geographic ecological momentary assessment (GEMA)–leveraging JITAI that incorporates trauma-informed principles and personalizable geofencing to meet the needs of people with HIV. We report the findings of the pilot of NOLA (New Orleans, Louisiana) Gem, a JITAI adaptation of LIFT, trialed among a sample of people with HIV enrolled across Greater New Orleans. The NOLA Gem app was adapted from the *Living in the Face of Trauma: An Intervention for Coping with HIV and Trauma* manual, in consultation with Sikkema [27,28]. The NOLA Gem study covered Aims I to III. The findings of Aim I are reported in detail in Gasik et al [21]. The results of Aim II, the NOLA Gem design synthesizing the insights of Aims I and II (including a detailed user flow diagram and user story maps), and the pilot trial protocol are described in Kabir et al. Here, we report the results of Aim III.

## Methods

The NOLA Gem Aim III study was a randomized controlled trial enrolling adults with HIV across Greater New Orleans, spanning from 2023 to 2024.

### GEMA: Control Arm

The NOLA Gem app was designed with a control and a treatment version, which were masked to the user. The assignment was dependent on how the user was created in the admin panel by research staff. Both versions had GPS tracking; latitudinal and longitudinal data points were recorded at 1-minute intervals if the device moved more than 20 m for the duration of the 21-day pilot trial [43]. GEMA alone, combining GPS tracking and daily diary delivery via text message at 9:00 AM (due by 12:00 PM) and 6:30 PM (due by 9:30 PM) intervals, but lacking any interventional component, constituted the control condition.

### NOLA Gem + GEMA: Treatment Arm

The treatment arm of the NOLA Gem app leveraged the GEMA inputs as tailoring variables, calling on fixed decision rules determined in Aim II, to recommend from among 7 “just-in-time” adaptive coping skills (“Set a SMART Goal,” “Common Irrational Thoughts,” etc) mapped to predetermined proximal outcomes [39,44]. Each skill was adapted from a LIFT worksheet, culturally tailored to invoke drivers of resilience identified by New Orleanian people with HIV in Aim II. Endorsing more than “a little stress” on daily diary 37f (“Since your last diary, how much stress did you feel today from any of the following? *Had an argument with friend, family member, or romantic partner*”) elicited deep-linked recommendations for the “Adaptive Coping” and “Common Irrational Thoughts” in-app brief skills modules, to give just one example. In addition, NOLA Gem users were provided 7 self-directed sessions (estimated 4‐13 minutes in duration), grounded in the traditional, linear delivery of LIFT. These sessions offered psychoeducation on maladaptive or adaptive coping strategies, goal setting, and mindfulness meditation, among other topics. As no generalizable across-case geospatial stressors were evident in Aim I [21], the NOLA Gem treatment arm included a user-determined, essentially personalizable, geofencing feature. Users were asked at baseline to identify 3 locations that they found to be particularly stressful in their lives. Latitude and longitudinal coordinates for these locations were entered into the admin panel with a 150-m geofence; when users entered this geofence, they received a text message asking if they would like to practice their skills, with a link to the skills section of the app. The NOLA Gem JITAI features, built atop and including all aspects of GEMA, constituted the treatment condition for the duration of the 21-day trial period.

**Figure 1.**
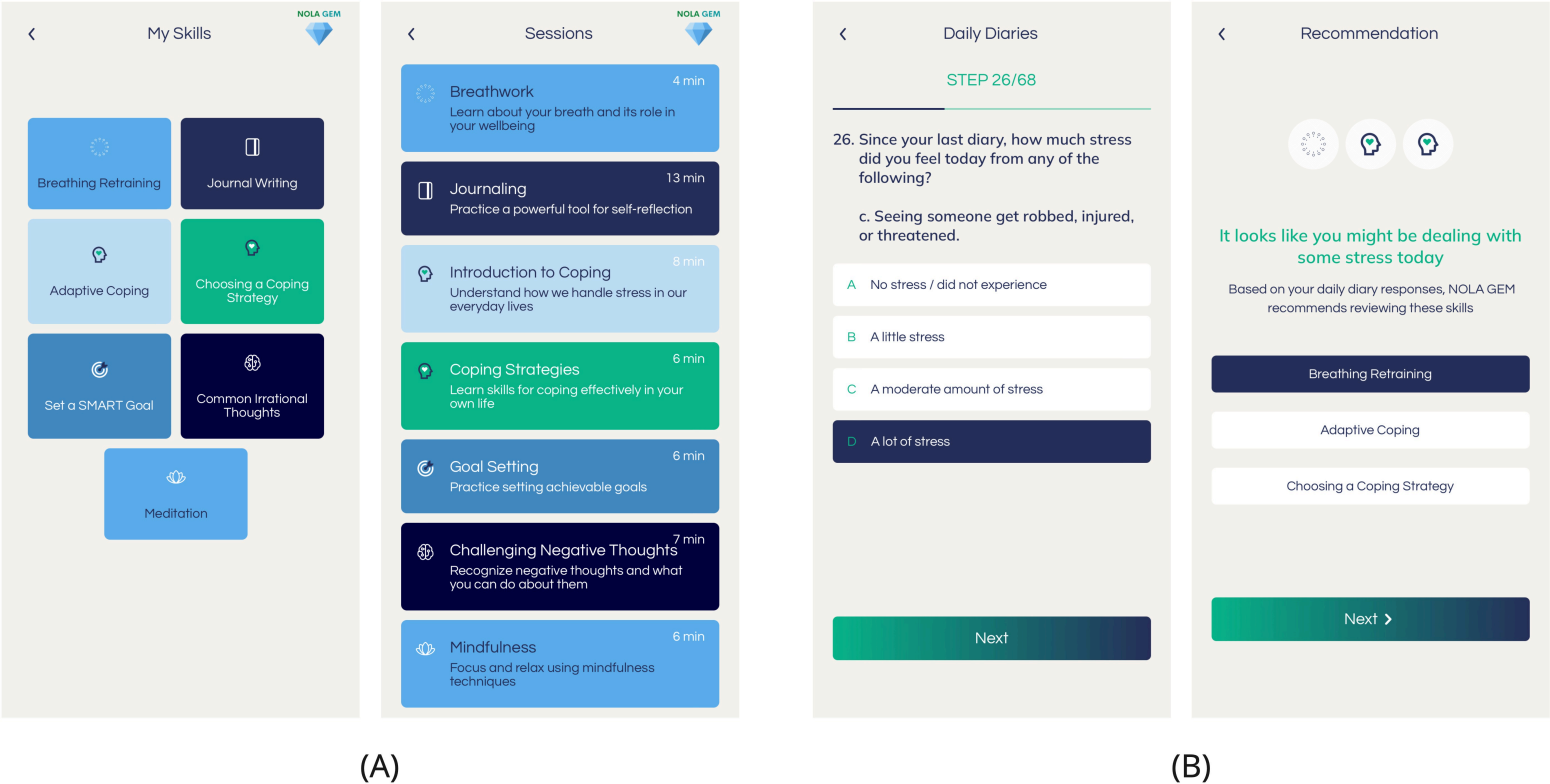
The New Orleans, Louisiana (NOLA) Gem + GEMA user interface: panel (A) displays the “My Skills” and “Sessions” menus; panel (B) displays a sample daily diary and the resultant skills recommendation prompt. GEMA: geographic ecological momentary assessment.

Illustrative screenshots of NOLA Gem are shown in Figure 1.

In both arms, the study app was Health Insurance Portability and Accountability Act–compliant and transmitted all data to a Health Insurance Portability and Accountability Act–compliant, Crowdbotics-hosted administrator dashboard.

### Study Participants and Procedures

Participants for Aims I and II were convenience-sampled from the longitudinal New Orleans Alcohol Use in HIV cohort, which, since 2017, has followed 365 people with HIV and hazardous alcohol use histories in outpatient care across Greater New Orleans [45]. For Aim III, this study enrolled participants from this cohort while diversifying recruitment to include a range (n=12) of local HIV outpatient clinics and community organizations serving people with HIV, which were provided with study recruitment flyers.

Participants were eligible if they (1) were 18 years or older; (2) maintained a current ART prescription, verified in person or via a photograph uploaded to a secure Qualtrics link; (3) owned a smartphone; and (4) indicated experiencing at least 1 incident of violence on the screener.

Study enrollment spanned from October 2023 to August 2024. Initially, eligible, consented participants were randomly assigned 1:1 to treatment (NOLA Gem + GEMA) or control (GEMA) arms. Once total enrollment reached 16, the remaining participants were assigned to the treatment arm. This step was taken to address compounding enrollment challenges posed by the ongoing COVID-19 pandemic, specifically the Delta and Omicron variants (during the former wave, Louisiana recorded the highest number of new cases relative to the total population in the United States [46]), and Hurricane Ida, which caused widespread evacuations and a prolonged power outage across Orleans Parish [47]. By favoring treatment-arm enrollment, we aimed to achieve a sufficiently sized pilot sample to gather preliminary mixed methods evidence of NOLA Gem’s acceptability, feasibility, and usability, despite these unforeseen obstacles, while maximizing the potential human benefits of research involvement for people with HIV in New Orleans.

The study arm was randomly assigned following eligibility verification, prior to the baseline appointment, to streamline onboarding. At the baseline appointment, participants were read an informed consent form aloud, and verbal consent was recorded in Research Electronic Data Capture (REDCap). The NOLA Gem app was then downloaded from the App Store (iOS) or Google Play (Android) onto participants’ phones using the institutional Wi-Fi. The research assistant enabled notifications and location tracking on participants’ devices, as well as location services for the NOLA Gem app. Onboarding to both control and treatment conditions was supported by illustrated guides. The baseline assessment was research assistant–administered, and responses were recorded in REDCap.

### Measures

#### Baseline Assessment

Demographics were collected at baseline: name; date of birth (to verify self-reported age); address, including ZIP code; assigned sex; gender (male, female, nonbinary, and a “prefer to self-describe” open-ended response option); race, using US Census categories along with a self-describe option; Latine heritage, using a single-item indicator; and sexual orientation (heterosexual or straight, gay or lesbian, bisexual, or self-describe). Relationship status, educational attainment, employment, and housing details were collected using measures carried over from the NOAH parent study [48]. Participants were administered a 4-item Digital Confidence Score measure (eg, “I use a digital device frequently”) adapted from Benson [49].

At baseline, a range of clinical variables were captured. Measures used in these analyses, which exploratorily probed potential drivers of, or disparities in, NOLA Gem acceptability, included the 3-item Alcohol Use Disorder Identification Test–Consumption, administered to detect potentially hazardous alcohol use patterns [50]; the Addiction Severity Index “Alcohol/Drugs” section, which collected past 30-day frequency of alcohol, cannabis, cocaine or crack, opioid, other, and combined substance use or misuse [48]. The PTSD Checklist for *DSM-5* assessed subthreshold and PTSD symptoms that may benefit from treatment [51]. The Posttraumatic Growth Inventory evaluated positive within-person aftereffects of traumatic stress, such as deepened spirituality and enhanced appreciation of life [52]. The “lost friends due to serostatus,” “disclosure risk,” “interpersonal discomfort,” and “hurtful attitudes toward PWH” items of the HIV Stigma Scale assessed those domains of internalized HIV stigma [53]. The 10-item Kaiser Permanente ACEs Questionnaire collected instances of early-life adversity, neglect, and abuse [54].

#### Quantitative Postintervention Assessment

At offboarding, both GEMA and GEMA + NOLA Gem users were administered a range of acceptability and usability measures. These end points (preregistered in Skeen et al,[55] crucial for the future trialability and scalability of NOLA Gem, are the focus of this study.

Acceptability was assessed using a series of study-specific items designed to evaluate ease of use, perceived success in enhancing well-being, and privacy concerns. For example, the first item asked, “How often were you able to open and use the NOLA GEM app without confusion, frustration, or asking for help? [Very often, somewhat often, not very often].” Ten items, using a 4-point Likert scale, assessed the NOLA Gem app’s multifactorial appeal (eg, likeability, visual appeal), and its perceived helpfulness in attaining proximal outcomes; for example: “I found NOLA Gem to be helpful in changing habits [Agree, somewhat agree, somewhat disagree, disagree].” In addition, to test whether participants were willing to expend their own social capital on behalf of NOLA Gem, an adapted Net Promoter Score (NPS) asked participants about their likelihood of recommending NOLA Gem to a friend (1=not at all likely, 10=extremely likely) [56].

Feasibility was measured via attrition rates postenrollment, daily diary completion rates, and paradata descriptives.

Usability was assessed with the 8-item User Experience Questionnaire (UEQ)–*Short*, an abbreviated version of the UEQ that encompasses both *pragmatic* (determinative of user goals) and *hedonic* (determinative of user pleasure) usability domains. The UEQ uses a 7-point scale with item-specific anchors to deter central tendency biases [57]. For accessibility’s sake, we simplified these anchors wherever possible. For example, we replaced “obstructive” with “unhelpful” in item 1: “Rate the app from unhelpful to supportive.”

#### Qualitative Postintervention Assessment

Finally, participants were asked a series of open-ended, atheoretical questions: “What did you not like about the app?” “What were your favorite parts of the app?” “What aspects of the app do you think should be changed?” and “What aspects of the app do you think should be kept?” Responses were digitally recorded and transcribed verbatim into dedicated REDCap fields.

### Ethical Considerations

All procedures were reviewed and approved by the Socio-Behavioral Institutional Review Board of the Biomedical and Social Behavioral Institutional Review Boards at Tulane University (reference number 2022‐1861). All participants provided verbal informed consent. All recorded data were private, confidential, and anonymous. Participants were compensated with a US $30 Amazon gift card for completing their baseline appointment, US $20 via ClinCard for their immediate postassessment and 30-day follow-up visit (each), US $2 via ClinCard for each daily diary completed, and a US $10 Walmart gift card for the 90-day follow-up. Starting in March 2024, after switching to treatment-arm only enrollment, participants were also compensated with US $5 via ClinCard per session completed, with a US $15 bonus if all 7 were completed.

### Analytic Approach

#### Overview

Background demographic and quantitative acceptability, feasibility, and usability outcomes are reported descriptively. Although the UEQ “Data Analysis Tool” provides a benchmark usability dataset, these values are not validated for the 8-item UEQ short form [58]. Disparate acceptability was assessed, preliminarily, by recoding dichotomous gender (1=male), race (1=Black), Latine identity (yes=1), Test–Consumption and Addiction Severity Index item-wise (1=any lifetime alcohol or past 30-day cannabis use, cocaine or crack, or opioid use), dichotomizing the PTSD Checklist for *DSM-5* at the recommended >33 cutoff, and median-splitting the Post-Traumatic Growth Inventory and *Digital Confidence Score* sum, for entry into bivariate logit regressions as factor variables [59]. Wherever sufficient variation permitted, acceptability outcomes were dichotomized as well (eg, 1 = “Agree” or “Somewhat agree” to “I found NOLA Gem to be useful”). These transformations were undertaken to account for low variability and a very small sample size.

Particular attention was paid to possible differential drivers of GEMA privacy concerns (recoded such that 1=“somewhat” and “very concerned” [60]). Potential privacy concern predictors included sexual orientation (1=sexual minority), and median-split HIV Stigma Scale and *ACEs* scores.

The NPS was computed in the industry-standard manner: scores ≥9 were categorized as *promoters*, scores 7 to 8 as *passives*, and scores ≤6 as *detractors*. The NPS metric was obtained by subtracting the percentage of detractors from the percentage of promoters [56].

While direct control-treatment differentials in acceptability (etc) were not preregistered, we tested for potential GEMA (control) versus NOLA Gem + GEMA (treatment) additive interaction costs exploratorily, via 2-tailed independent-samples *t* test and visual inspection, where warranted [61]. Quantitative analyses were run in Stata/SE 18.0 [62]. Data visualizations were created using Naqvi spider package for Stata [63] and Matplotlib in Python 3.13 [64].

Qualitative responses were analyzed using a rapid, atheoretical, human–large language model (LLM) synergistic approach. First, the lead author (SJS), after conducting immersive close readings of the treatment group responses, developed categories inductively, and formalized them within a codebook. The qualitative data frame was transformed by inserting category-specific columns to (1) dummy code each response for every respective category, as warranted, and (2) extract the most salient segment of text: the “rationale” for the coding application [65]. Next, informed by the work of Dunivin et al [66] and the CHALET framework of Meng et al [67], the “Collaborative Human-LLM Analysis for Empowering Conceptualization in Qualitative Research” technique was used. Category-by-category deductive coding prompts were formulated. The prompts were “few-shot,” providing human-synthesized examples, and leveraged contemporary LLMs’ chain-of-thought capabilities by requiring a 2-sentence justification for each coding decision [66,67]. To maintain data privacy, the Ollama framework was used to run a local on-device 12 billion–parameter (12B) Gemma 3, a lightweight open-source extension of Google’s proprietary Gemini foundation model [68,69]. Matching the initial human approach, the 12B Gemma 3 was tasked with (3) dummy coding expressions of each category and (4) providing coding decision justifications (inclusive of the LLM’s own “rationales”) in dedicated columns.

Initial human-LLM intercoder reliability was computed using Cohen *κ*. For any category that demonstrated *κ* <0.70, a second human consensus coder (ST) resolved coding application discrepancies. All qualitative analyses were conducted in Python 3.13.

#### Open Code

Stata .do and Python .ipynb replication scripts, including deductive coding prompts, are hosted in a dedicated GitHub repository [70].

## Results

### Overview

The overall pilot sample was generally in their mid- to late-50s, with slight over-representation by men (n=17, >55%), a plurality identifying as minoritized sexualities (n=10, <45.45% heterosexual/straight), and roughly 3 quarters Black (n=22, 73.33%). A very large majority (n=30, 93.33%) reported <US $50,000 past-year household income. Digital confidence scores were high, in the “strongly agree”–“agree” range on average.

The pilot sample’s demographic details, overall and by study arm, are shown in Table 1.

**Table 1.** Baseline sociodemographic attributes and digital confidence: NOLA^a^ Gem pilot.

Attribute	Descriptives^b^		
	Overall (N=30)	Treatment: GEMA^c^ + NOLA Gem (n=22)	Control: GEMA (n=8)
Age (y), mean (SD; range)	54.2 (11.9; 28‐74)	51.8 (12.6; 28‐74)	60.9 (6.06; 50‐72)
Gender, n (%)^d^			
Male	17 (56.67)	15 (68.18)	2 (25.00)
Female	13 (43.33)	7 (31.82)	6 (75.00)
Nonbinary	0 (0)	0 (0)	0 (0)
Preferred to self-describe^e^	0 (0)	0 (0)	0 (0)
Sexual orientation, n (%)^d^			
Heterosexual or straight	13 (43.33)	10 (45.45)	3 (37.50)
Gay or lesbian	11 (36.67)	9 (40.91)	2 (25.00)
Bisexual	3 (10)	1 (4.55)	2 (25.00)
Preferred to self-describe^e^	3 (10)	2 (9.09)	1 (12.50)
Ethno-racial identification, US Census categories, n (%)^d^			
African-American/Black	22 (73.33)	15 (68.18)	7 (87.50)
Asian	0 (0)	0 (0)	0 (0)
Multiracial	3 (10)	2 (9.09)	1 (12.50)
Native Hawaiian or Pacific Islander	0 (0)	0 (0)	0 (0)
White	4 (13.33)	4 (18.18)	0 (0)
dta^f^	1 (3.33)	1 (4.55)	0 (0)
Hispanic or Latino	2 (6.67)	2 (9.09)	0 (0)
Household income in the past year (US $), n (%)^d^			
<10,000	6 (20)	4 (18.18)	2 (25.00)
10,000-19,999	13 (43.33)	8 (36.36)	5 (62.50)
20,000-34,999	3 (10)	2 (9.09)	1 (12.50)
35,000-49,999	6 (20)	6 (27.27)	0 (0)
50,000-74,999	2 (6.67)	2 (9.09)	0 (0)
75,000-99,999	0 (0)	0 (0)	0 (0)
>100,000	0 (0)	0 (0)	0 (0)
Housing, n (%)^d^			
Stand-alone single-family home	14 (46.67)	10 (45.45)	4 (50)
Apartment, duplex, or multifamily home	13 (43.33)	10 (45.45)	3 (37.50)
Group home or HIV-tailored group facility	1 (3.33)	1 (4.55)	0 (0)
Homeless or shelter	1 (3.33)	0 (0)	1 (12.50)
Nursing home	0 (0)	0 (0)	0 (0)
Preferred to self-describe^e^	1 (3.33)	0 (0)	1 (4.55)
Smartphone OS^g^, n (%)^d^			
Android	18 (60)	12 (54.55)	6 (75.00)
iOS	12 (40)	10 (45.45)	2 (25.00)
Digital confidence scores, mean (SD)^h^			
“I use a digital device frequently”	3.53 (0.73)	3.59 (0.73)	3.38 (0.74)
“Most of my friends use digital devices”	3.63 (0.49)	3.73 (0.46)	3.38 (0.52)
“I can usually get help if I am stuck”	3.37 (0.72)	3.41 (0.80)	3.25 (0.46)
“I feel confident using most digital devices”	3.17 (0.83)	3.27 (0.83)	2.88 (0.83)

a NOLA: New Orleans, Louisiana.

b Percentages may not total 100 due to rounding.

c GEMA: geographic ecological momentary assessment.

d Column percentages by attribute are shown.

e Details withheld to preserve participant anonymity.

f dta: decline to answer.

g OS: operating system.

h Adapted from Benson [49]; scored on a 4-point scale: 4=strongly agree, 3=agree, 2=neutral, 1=disagree.

### Acceptability

Acceptability, similarly, was generally very high across measures in the NOLA Gem + GEMA (treatment) group (n=22). Overwhelmingly, 16 (72.73%) participants reported the easy use of the app, with perceived helpfulness more equivocal, but a very small minority (n=2, 0%‐9.09%) perceiving the app as totally ineffectual. Bivariate logit regressions were null (male, *P*=.40; Black, *P*=.75; Latine, *P*=.26; lifetime alcohol use, *P*=.70; past 30-day drug use, *P*=.29; positive PTSD screen, *P*=.07; >median posttraumatic growth, *P*=.15; >median digital confidence, *P*=.22), suggesting no detectable social patterning to NOLA Gem acceptability among the pilot sample.

Nearly a third (n=7, 31.82%) reported GPS-related privacy concerns, though no statistically significant associations with privacy-related concerns were evidenced (male, *P*=.23; minoritized sexuality, *P*=.70; >median HIV stigma, *P*=.70; >median adverse childhood experiences, *P*=.30; as GEMA was universal, these models used the complete sample, ie, n=30). Consistent with the qualitative findings, nearly a quarter (n=7, 23.33%) found the daily diaries difficult to incorporate into their daily lives. Within the treatment group, 11 activated a custom geofence; though enthusiasm for this feature was mixed, no participants found it “not at all” helpful.

The app usage and satisfaction measures are summarized in Table 2.

**Figure 2.**
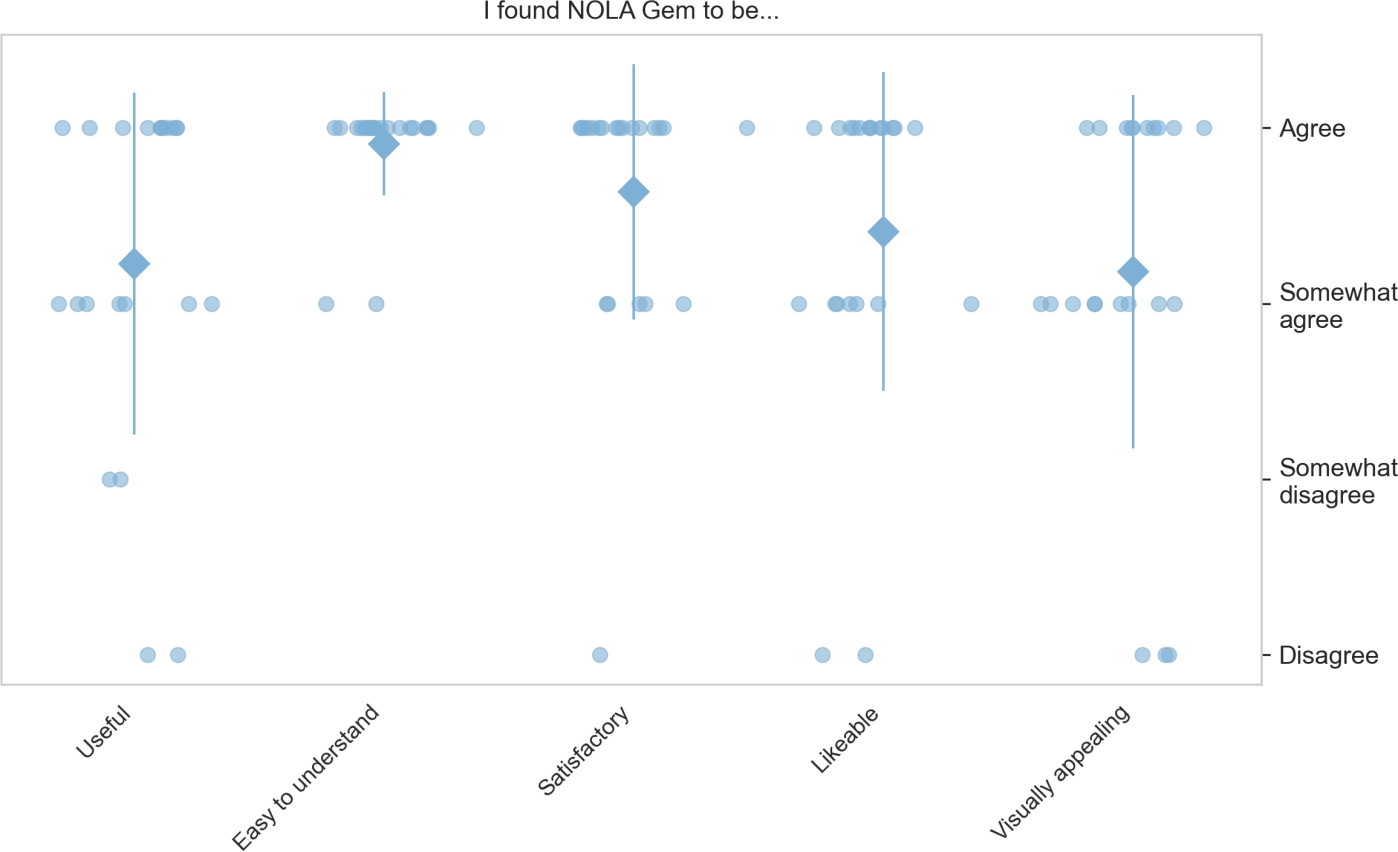
NOLA Gem acceptability: domain general. Diamond markers denote mean ratings, and error bars indicate SDs. NOLA: New Orleans, Louisiana.

Additional measures of multifactorial appeal are plotted in Figure 2.

**Figure 3.**
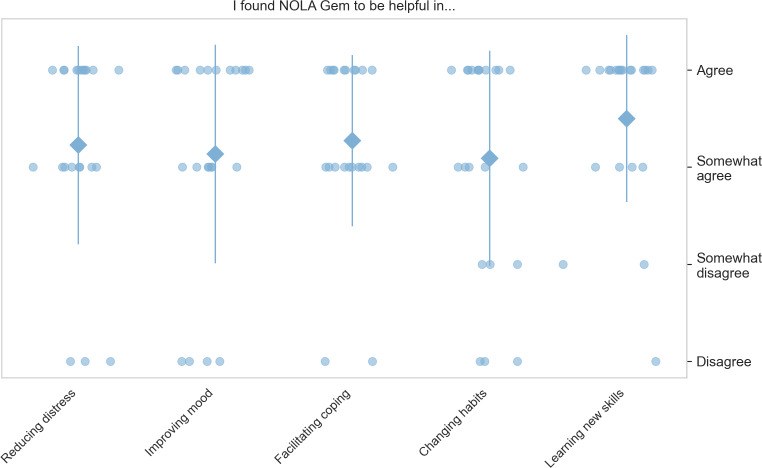
NOLA Gem acceptability: perceived helpfulness. Diamond markers denote mean ratings, and error bars indicate SDs. NOLA: New Orleans, Louisiana.

Measures of NOLA Gem’s perceived helpfulness are plotted in Figures3  4.

**Table 2.** App usage and satisfaction: geographic ecological momentary assessment (GEMA) + NOLA^a^ Gem (treatment group, n=22).

Measure	Response options, n (%)^b^	Mean (SD; range)
How often were you able to open and use the NOLA Gem app without confusion, frustration, or asking for help?	Very often: 16 (72.73) Somewhat often: 3 (13.64) Not very often: 3 (13.64)	2.59 (0.73; 1‐3)
How successful was the app in addressing your daily life?	Very successful: 11 (50) Somewhat successful: 11 (50) Not very successful: 0 (0)	2.50 (0.51; 1‐3)
How much has the app increased well-being, sense of calm, or positive feelings?	A lot: 11 (50) A little: 9 (40.91) Not at all: 2 (9.09)	2.41 (0.67; 1‐3)
Did you feel concerned about your privacy while participating in the GPS tracking part of this study?^c^	Not concerned: 15 (68.18) Somewhat concerned: 5 (22.73) Very concerned: 2 (9.09)	2.67 (0.61; 1‐3)
Did you receive text messages encouraging or suggesting skills or sessions to try in the NOLA Gem app?	Yes: 21 (70)	—^d^
If “yes” (n=21): How relevant were these to your daily life or stress?	Very: 2 (9.52) Somewhat: 10 (47.62) A little: 2 (9.52) Not at all: 1 (4.76) Did not use: 6 (28.57)	3.48 (1.17; 1‐4)
Did you receive suggested skills at the end of your daily diaries to try in the NOLA Gem app?	Yes: 21 (70)	—
If “yes” (n=21): How relevant were these to your daily life or stress?	Very: 7 (33.33) Somewhat: 10 (47.62) A little: 4 (19.05) Not at all: 0 (0) Did not use: 0 (0)	3.14 (0.73; 1‐4)
Geofencing alerts were text messages that appear when you neared areas you identified as stressful at the start of the study. Did you receive any geofencing alert text messages during the study period?	Yes: 11 (50)	—
If “yes” (n=11): How helpful were these recommendations in the moment?	Very: 5 (45.45) Somewhat: 3 (27.27) A little: 2 (18.18) Not at all: 0 (0) Did not use: 1 (9.09)	3.45 (0.93; 1‐4)
Was the timing of the daily diaries easy for you to incorporate into your daily life?	No: 7 (23.33)	—
(If “no”) Which timeframe was difficult for you to answer within?	9:00 AM-12:00 PM: 4 (57.14) 6:00 PM-9:00 PM: 0 (0) Both timeframes: 3 (42.86)	—

a NOLA: New Orleans, Louisiana.

b Percentages may not add to 100 due to rounding.

c Reverse coded.

d Not applicable.

**Figure 4.**
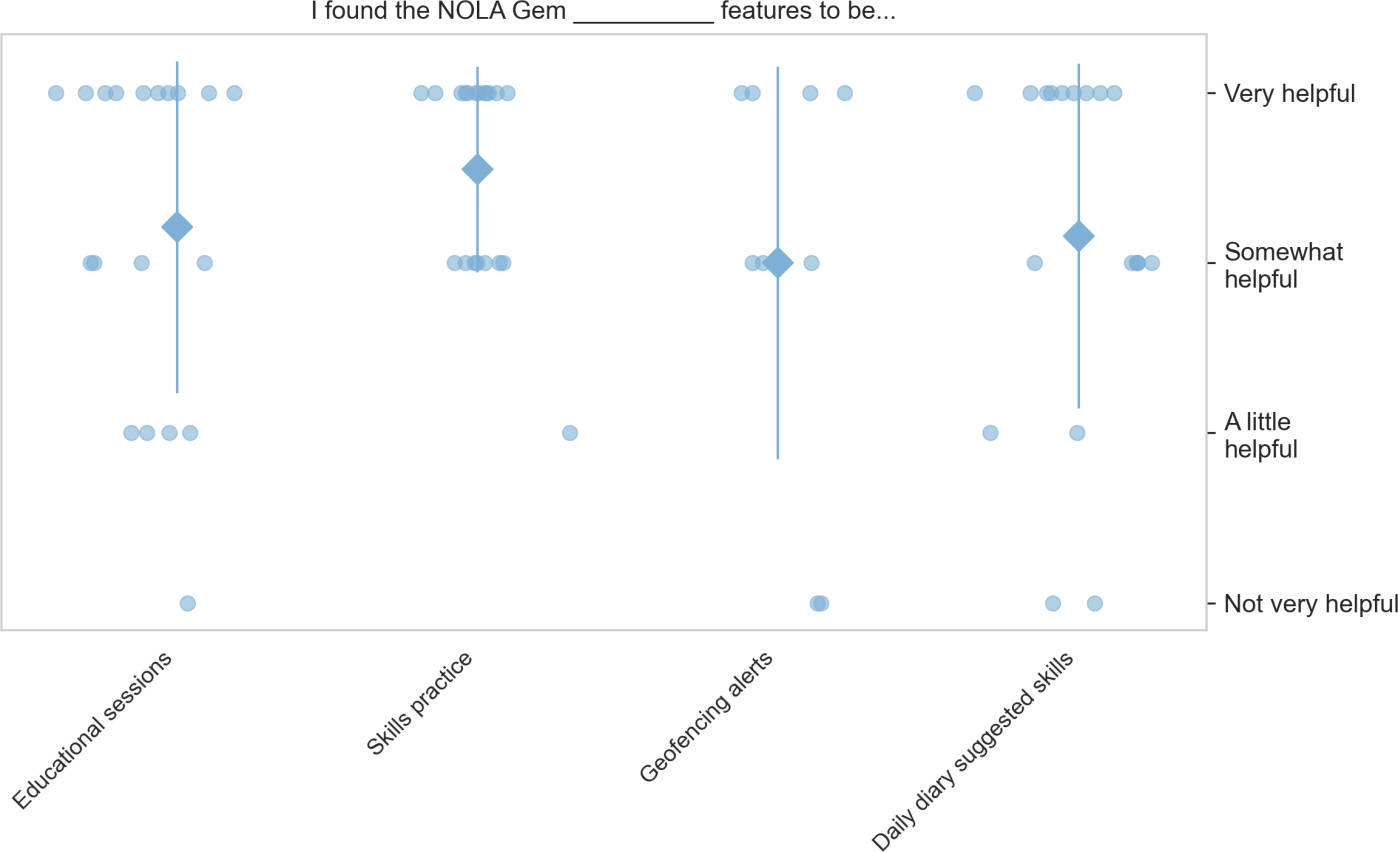
NOLA Gem acceptability (control): perceived helpfulness. Diamond markers denote mean ratings, and error bars indicate SDs. NOLA: New Orleans, Louisiana.

Because the control-treatment comparison was essentially additive, building features atop the already-demanding GEMA deployment, we tested for between-group differences in acceptability measures. Although, descriptively, GEMA measures tended to be higher (eg, treatment: mean 2.59, SD 0.73 vs control: mean 2.75, SD 0.46 for “able to open the app without confusion”), between-group *t* tests were uniformly null (*P*=.13‐.87).

NPS scores averaged (mean 7.70, SD 3.04). *Promoters* comprised 63.64% (n=14), and *detractors* comprised 22.73% (n=5) of the GEMA + NOLA Gem treatment sample, yielding an NPS metric of 40.91, approaching the generally accepted ≥50 “excellent” score [56].

### Feasibility

At baseline, 32 participants were enrolled, with 30 (93.75%; this study’s analytic sample) completing the 21-day trial period and completing ≥1 daily diary. Sociodemographic attributes of noncompleters are withheld to maintain participant confidentiality.

NOLA Gem paradata, collected from the treatment arm, showed that in a majority of the 607 completed daily diaries, across all participants (n=22), no skill was recommended: 463 (76.28%). Only 3 (13.64%) of the users completed 0 skills, and in 1 instance, technical errors may have prevented data recording.

These usage distributions are shown in Table 3.

**Table 3.** Living in the Face of Trauma (LIFT) skills: recommendations, time spent, completions: geographic ecological momentary assessment (GEMA) + New Orleans, Louisiana (NOLA) Gem (treatment group, n=22).

Skill	Recommendation frequency, n (%)^a^	Minutes spent		Completion frequency, n (%)^b^
		Count of 0-minute users, *n*_0_	Median_>0_ (range_>0_)	
Adaptive coping	14 (9.72)	14	10.0 (1‐409)	26 (12.68)
Breathing retraining	46 (31.94)	12	6.0 (1‐13)	46 (22.44)
Choosing a coping strategy	15 (10.41)	17	3.5 (1‐15)	30 (14.63)
Common irrational thoughts	14 (9.72)	16	3.0 (1‐31)	27 (13.17)
Journal writing	14 (9.72)	17	36.0 (7‐137)	16 (7.80)
Meditation	31 (21.53)	12	7.0 (2‐59)	41 (20)
SMART^c^ goal-setting	10 (6.94)	15	8.0 (1‐74)	19 (9.27)

a Calculated as *n* skill-specific recommendations/144 total skills recommendations.

b Calculated as *n* skill-specific completions/205 skills completions; completions>recommendations due to user-determined “pull” (nonprompted) skills practice.

c SMART: specific, measurable, achievable, realistic, and able to be achieved in time.

### User Experience

#### Overview

The short-form UEQ yielded generally good scores for NOLA Gem across the board, particularly on the “Supportive/helpful” (mean 5.82, SD 1.47), “Easy” (mean 5.91, SD 1.93), and “Efficient” (mean 6.14, SD 1.36) criteria. UEQ scores were qualitatively similar across conditions, suggesting negligible additional burden associated with the NOLA Gem components relative to GEMA alone.

**Figure 5.**
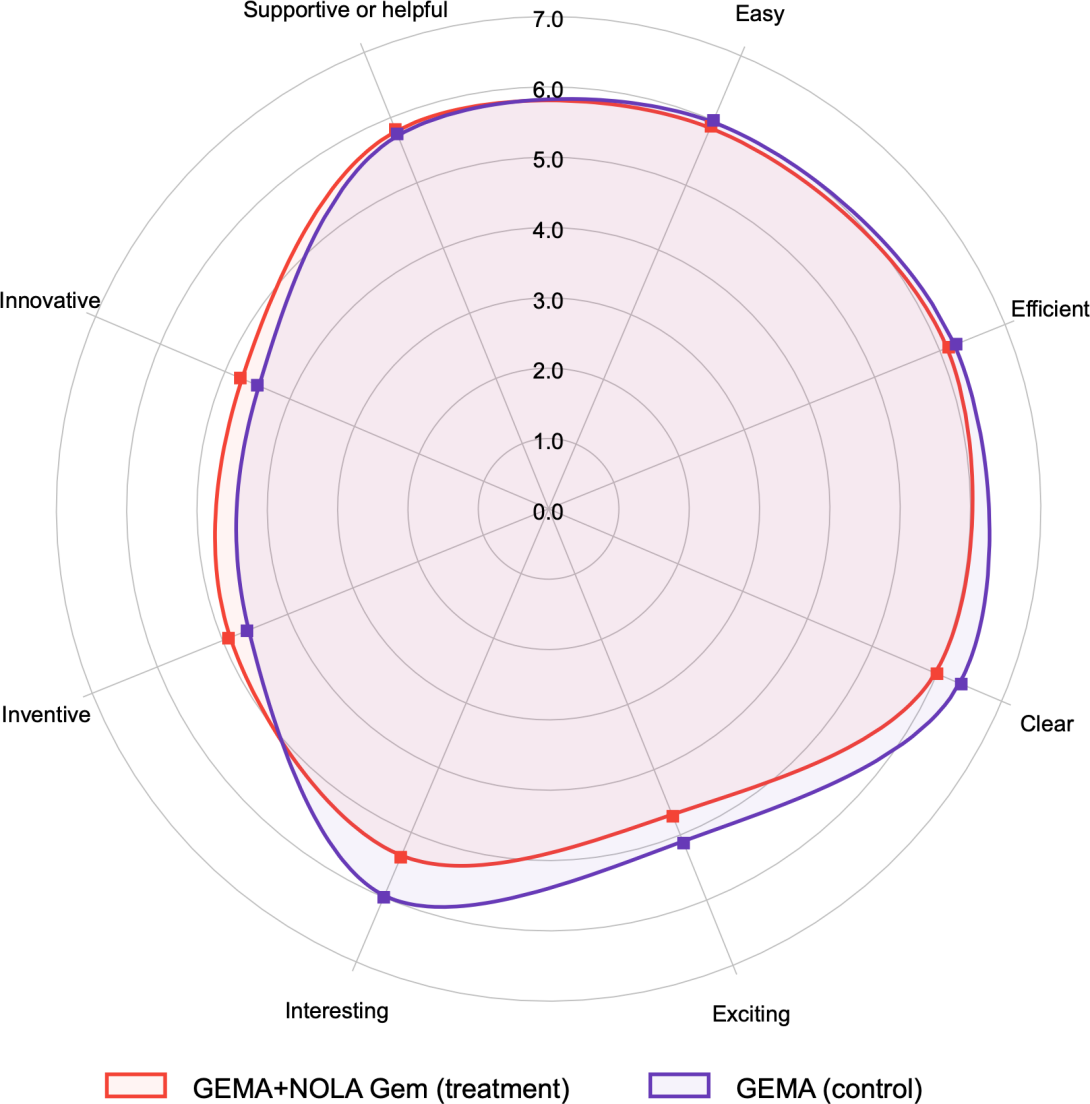
Short-form User Experience Questionnaire (UEQ) mean scores spider plot [63]: geographic ecological momentary assessment (GEMA) (control) versus New Orleans, Louisiana (NOLA) Gem + GEMA (treatment).

The distribution of these scores by study arm is visualized in Figure 5.

#### Qualitative Description

When prompted, many participants were able to specify their favorite NOLA Gem features. These favorites did not, however, exhibit any across-case consistencies: “I like how it was designed, the colors were very calming … It was simple and it was easy to navigate” (participant C); “The meditation, the extra sessions, the definitions. I read everything and it was very reassuring and inventive” (participant K). Some participants intuited the “just-in-time” EMA-triggered mechanisms of action, as captured by a “You seem a little stressed … it will tell you what you can do” in vivo code: “[I liked the app] asking about your health and your condition … When it’s checking up on your skills based on how you feel at one point in time in your daily diary of what you’re doing, depending on what space you’re in at the time” (participant I); “[I liked] the way when it asks you a question you seem a little stressed today it will tell you what you can do to relieve stress. I liked that” (participant R). Participant L described benefiting from a broad range of intervention components:

*I had a better [understanding] of myself, meditation and breath control, I really enjoyed all parts of the app and most of the time I got satisfactory. Men don’t talk and think about their feelings and I really liked how it brought my feelings to the surface. When I do meditation, thanks to your app I learned that I don’t have to be professional, I can just do it how it works for me. It also played a role in increasing my self-esteem. I never practiced breathing but thanks to your app I learned how to do it through the stomach.*

These benefits sometimes came with inherent tradeoffs, as pointed out by participant C:

*My favorite part is the different type of coping mechanisms that are offered. It will be great for those who need them, if they can get past the survey.*

Six persistently evident across-case topics were detected amid the qualitative responses, 4 subordinate to an overarching “Frictions and frustration” category. Cohen *κ* scores, by code, ranged from 0.49 to 1.00. As such, human-LLM coding applications of “EMA questionnaire length [*κ*=0.64],” “EMA prompt timing [*κ*=0.63],” “EMA item attunement [*κ*=0.68],” and “You seem a little stressed … it will tell you what you can do [in vivo; *κ*=0.49]” underwent additional human triangulation and review to achieve consensus.

The length and timing of the daily diary surveys were consistently mentioned as frustrations among NOLA Gem participants: “The number of questions. Unable to complete the questions in three hours. Inconvenience part of the three-hour time limit” (participant E); “Survey is too long and redundant. No reason to ask the same questions in a different way” (participant F). Participant E pointed to the daily conflicts potentially raised by study participation (and, by extension, intervention fidelity): “Being that I work on a boat that starts at 6 and doesn’t dock until 10, I do not have time to complete the entries”; “The times for diaries were inconvenient due to my work schedule,” participant P concurred. The daily diary’s *attunement* to the lives of enrolled people with HIV, as well as each question’s cultural congruence and ecological validity in tapping routine stressors, was questioned sporadically as well: “the questions were all basically the same, in some cases it did not include emotions that I thought should be included … it asked about seeing trash in the neighborhood and I don’t really understand how is that relevant for daily life” (participant J); “I do not drink and I could not answer the questions. Did not feel the questions applied to everyday life experiences for PWH” (participant H). In addition, occasional malfunctions were experienced by certain users: “I don’t like that it glitches, last time I used it, it wasn’t glitching, this time you could not log in man I didn’t like that” (participant G).

Although some participants described the app’s “perfection” (“Nothing is going to be perfect, but this app is perfect. All the tools. Keep it authentic, just the way you designed it,” participant L), various recommendations for updates were elicited. These included multimedia options: “… incorporating audio to where it’s like soothing or calming white noise, ocean, waterfalls, stuff like that” (participant C). Participant J felt that opportunities remain to activate the *adaptive* aspect of JITAI design: “I think that it should be a little more interactive, more interactive and it should adjust to what questions are being asked … doesn’t seem to be lifelike; say after it asked about being depressed, it would just move on to the next question rather than going deeper into it.” Similarly, participant P saw opportunities for enhanced personalization of the geofencing feature: “Just ask if a person is okay with being reminded that they’re in a stressful area before they get alerted about it being stressful if you didn’t ask beforehand.”

## Discussion

### Principal Findings

We trialed NOLA Gem, a trauma-informed JITAI leveraging GEMA inputs to provide people with HIV in New Orleans adaptive coping skills refreshers, against a GEMA-exclusive control, over a 21-day pilot period. In general, GEMA + NOLA Gem users (n=22) endorsed high acceptability, particularly in domains of perceived helpfulness, ease of comprehension, and ease of use. Of the 32 participants enrolled, 30 (93.75%) completed at least 1 daily diary, highlighting the app’s feasibility. Among GEMA + NOLA Gem (treatment-arm) participants, for every skill, completions exceeded recommendations, indicating a nontrivial rate of “pull,” or user-determined, app interactions, and suggesting valuable “Big E” (behavioral) engagement among a subset of dedicated users [39,44]. Most skills saw zero inflation among their “minutes spent” distributions, which were heavily skewed; this pattern might indicate user self-selection into accessible LIFT skills. Conversely, these users might miss out on new and potentially more beneficial coping skillsets. Qualitative user experience concerns converged on the frictions and interaction costs associated with the twice-daily EMA inputs: their sometimes-inconvenient timing and often-burdensome duration. While the relatively low skills recommendation rate *among* daily diary completers suggests those items may not be parsimoniously tapping frequently encountered stressors, the lower recommendation rates also prevent habituation—users “tuning out” a JITAI due to excess “push” notifications of no clear benefit [44]. Nevertheless, many participants declined to offer recommendations for next-generation changes, describing the app as “perfect” and acknowledging the value of the “just-in-time” adaptive coping skills delivery intuitively.

The *protocolization*, or defining the ruleset that determines how intraindividual dynamic inputs trigger just-in-time interventions [44], for each stressor-triggered adaptive coping skills prompt was formally mapped by the study team via feature hypothesis charting in 2022. Work on within-person JITAI optimization has advanced substantially since then, incorporating methods as diverse as reinforcement learning [71], *N*-of-1 model-predictive controller personalization algorithms [72], LLM-enabled free-text user preference updates in real time [73], and, most commonly, microrandomized controlled trials to isolate the most promising, in Nahum-Shani terms, “states of vulnerability/opportunity” and “states of receptivity” for momentary intervention delivery [74]. NOLA Gem, in contrast, was protocolized based on the findings of Aims I and II [21] and HIV biobehavioral domain expertise. These rulesets were static [55], even as they responded daily to EMA inputs. It is evident, based on our qualitative findings, that the sprawling battery of inflexibly scheduled daily diaries [55] was the primary driver of user burden and, as such, the foremost threat to acceptability. Despite some participants’ insistence that “nothing” in NOLA Gem should change, a clear opportunity exists to optimize the JITAI mechanisms of action, *re*-protocolizing and pruning surplus daily diary measures in an evidence-based manner.

Though, by most standards, our findings support the advancement to a fully powered randomized controlled trial, in light of the abovementioned emergent methods for JITAI optimization, an additional microrandomized controlled trial cycle using contemporary techniques may further enhance acceptability. Launching intraindividual microrandomized trials, the most robust *stressor → state of receptivity → coping skill → proximal outcome → distal outcome* dynamics can be modeled and protocolized, at a within-person and within-user base (eg, across an outpatient HIV clinic network) level [75]. Indeed, the required in-app feature set already exists, in the form of single-item “SkillCheck” quizzes tap each delivered skill’s success in improving a hypothesized JITAI-consistent proximal outcome.

Such improvements would benefit the prospective user base of people with HIV. However, the efficiencies built in by such a “pruning” cycle would benefit future clinical workflow integration—a potential prerequisite to a successful Phase III trial—and, ultimately, scalability and sustainment. The nonadoption, abandonment, scale-up, spread, and sustainability of technological innovation framework recognizes 7 domains. These include the condition, or health issue itself; the technology under consideration; its inherent value proposition, supply- or demand-side; adopters, including staff, patients, caregivers, and providers; organizational climate; political and regulatory considerations; and temporal aspects of implementation and organizational resilience (or lack thereof) [76]. Interventions that incorporate rampant complexity across the sum of these domains are shown, across empirical case studies and hermeneutic literature reviews [77], to rarely, if ever, achieve scale and sustainability. HIV prevention and treatment, despite past policy successes, remain subject to a volatile political climate [78]. People with HIV engaged in outpatient care endure poverty, violence, and medical and psychiatric comorbidities disproportionately [1,7,11,79]. Complexity, across the HIV continuum of care, is all but assured. Even at the pilot stage, it is incumbent upon technologists and HIV digital therapeutics investigators to prioritize simplicity, wherever possible, in intervention design and delivery. The NOLA Gem pilot study, and, in particular, the valuable insights contributed by NOLA Gem pilot users, lay the groundwork for concurrent optimization via simplification to advance next-generation HIV mobile health.

This study is subject to limitations as well. First, the pilot sample was not powered to detect between-group differences, either in GEMA versus GEMA + NOLA Gem acceptability (a proxy for differential interaction costs [61]), or predictors of disparate acceptability across subsets of outpatients with HIV. Null findings on these questions should be understood as highly preliminary. In particular, we are unable to extend the important work of Hubach et al [60], revealing EMA-related confidentiality concerns among a sample of rural men who have sex with men. A confluence of external factors—namely the COVID-19 variants still endemic in Louisiana, and Hurricane Ida [46,47]—delayed the trial launch substantially, hampering our ability to obtain our preregistered enrollment target of 60 participants, and to achieve balanced 1:1 random assignment. Though oversampling treatment-arm participants was ultimately to the benefit of these analyses, focused on the experiences of those NOLA Gem–assigned participants, our capacity to demonstrate equivocal acceptability across GEMA and JITAI paradigms is, similarly, undermined. We are also unable to gauge meaningful engagement metrics via paradata due to the small sample size, beyond a dichotomized “high” versus “low” engager subsetting [80]. Although these findings suggest future directions for NOLA Gem optimization, and support the need for a fully powered randomized controlled trial, attention toward protocolizing user-centered in-app adaptiveness to spur engagement is needed [45,75].

### Conclusions

Trauma, particularly trauma rooted in firsthand violence exposures, disproportionately affects the health and wellbeing of people with HIV [1]. Interventions tailored to the needs of people with HIV must not only address environmental stressors across spatial and temporal dimensions [17-20], but they must also embrace principles of trauma-informed care [2,7]. To this end, we developed NOLA Gem, a smartphone-delivered JITAI adaptation of the effective LIFT group intervention [39]. This pilot study demonstrated the acceptability, feasibility, and usability of NOLA Gem, supporting future optimization, and a fully statistically powered evaluation of the app’s efficacy among outpatients with HIV.

## Abbreviations

ACE: adverse childhood experience
EMA: ecological momentary assessment
GEMA: geographic ecological momentary assessment
JITAI: just-in-time adaptive intervention
LIFT: Living in the Face of Trauma
LLM: large language model
NOLA: New Orleans, Louisiana
NPS: Net Promoter Score
PTSD: posttraumatic stress disorder
REDCap: Research Electronic Data Capture
UEQ: User Experience Questionnaire
